# Two new species of the genus *Doryphorina* Melichar, 1912 (Hemiptera, Fulgoromorpha, Dictyopharidae) from China

**DOI:** 10.3897/zookeys.416.7498

**Published:** 2014-06-16

**Authors:** Yan-Li Zheng, Lin Yang, Xiang-Sheng Chen

**Affiliations:** 1 The Provincial Key Laboratory for Agricultural Pest Management of Mountainous Region / Institute of Entomology, Guizhou University, Guizhou Province, 550025 China; 2 Guizhou Normal College, Guizhou Province, 550018 China

**Keywords:** Dictyopharid, distribution, Fulgoroidea, planthopper, taxonomy

## Abstract

Two new species of the genus *Doryphorina* Melichar, 1912, *D. conglobatus* Zheng, Yang & Chen, **sp. n.** and *D. guizhouensis* Zheng, Yang & Chen, **sp. n.**, from China are described and illustrated. A key is given to identify all the known species of *Doryphorina*.

## Introduction

The dictyopharid planthopper genus *Doryphorina* (Hemiptera: Fulgoromorpha: Dictyopharidae) was established by Melichar (1912) based on a single species *Doryphorina stali* Melichar, from Sumatra. Later, Fennah (1978) described two subspecies *Doryphorina stali minor* and *Doryphorina stali subdeflexa* from Vietnam. Song and Liang (2013) revised and elevated the status of the two subspecies to species, the genus contained three species *Doryphorina stali* (Burma, Malaysia, Indonesia), *Doryphorina minor* (Vietnam, China: Guangxi, Hainan, Guangdong, Guizhou) and *Doryphorina subdeflexa* (Vietnam, China: Yunnan). In this paper, two new species *Doryphorina conglobatus* sp. n. and *Doryphorina guizhouensis* sp. n. from China, are described and illustrated. A key to identify all the known species of *Doryphorina* is given.

## Material and methods

Material examined here is deposited in the Institute of Entomology, Guizhou University, Guiyang, China (GUGC). Dry specimens were used for the observation, description and illustration. Genital segments of the examined specimens were macerated in boiling solution of 10% NaOH and drawn from preparations in glycerin jelly under a Leica MZ12.5 stereomicroscope. Color pictures for adult habitus were obtained by a KEYENCE VHX-1000 system. Illustrations were scanned with Canon Cano Scan LiDE 200 and imported into Adobe Photoshop CS6 for labeling and plate composition. Terminology of morphology, genital characters and measurements follow Song and Liang (2013).

The following abbreviations are used in the text, BL: body length (from apex of cephalic process to tip of fore wings); HL: head length (from apex of cephalic process to base of eyes); HW: head width (including eyes); FWL: forewing length; GUGC: Guizhou University, Guiyang, China.

## Taxonomy

### Doryphorina

Taxon classificationAnimaliaHemipteraDictyopharidae

Melichar, 1912

Figs 1 – 32

Doryphorina Melichar, 1912: 99. Type species: *Doryphorina stali* Melichar, 1912; by original designation.
Doryphorina : Schmidt 1915: 76; Distant 1916: 25; Schmidt 1928: 129; Metcalf 1946: 87; Fennah 1978: 254; Emeljanov 2011: 1125; Song and Liang 2013: 2.

#### Type species.

*Doryphorina stali* Melichar, 1912 (original designation).

#### Diagnosis.

For the relationships and a diagnosis of *Doryphorina* see Song and Liang (2013).

#### Distribution.

Oriental region.

#### Key to species of *Doryphorina* Melichar, 1912 based on males

(Modified from Song and Liang 2013)

**Table:** 

1	Vertex broad, lateral carinae nearly parallel; forewings without dull ochraceous spot near stigma	2
–	Vertex relatively narrow, broadest at base and apex, lateral carinae not parallel; forewings with a dull ochraceous spot near stigma	3
2	Dorsal apical lobes of phallobase with (Fig. 21) long and slender, with 3 long spines at base in dorsal view	*Doryphorina conglobatus* sp. n.
–	Dorsolateral apical lobes of phallobase directed laterally, with 2–3 long apical spines in dorsal view	*Doryphorina subdeflexa* Fennah
3	Cephalic process relatively short, with ratio length to length of pronotum and mesonotum combined less than or equal to 2.0	4
–	Cephalic process relatively long, with ratio length to length of pronotum and mesonotum combined 2.2	*Doryphorina guizhouensis* sp. n.
4	Gonostyles with upper process relatively short and broad; aedeagus with 2 pairs of apical lobes in ventral part	*Doryphorina minor* Fennah
–	Gonostyles with upper process distinctly slender and long; aedeagus with 1 pair of apical lobes in ventral part	*Doryphorina stali* Melichar

### Doryphorina conglobatus

Taxon classificationAnimaliaHemipteraDictyopharidae

Zheng, Yang & Chen sp. n.

http://zoobank.org/CEEAC18E-B365-4511-B226-77BAE4CE903E

Figs 1–5 , 11–21

#### Measurement.

♂, BL: 16.2 mm; HL: 4.6 mm; HW: 1.7 mm; FWL: 9.9 mm.

#### Description.

Body greenish-ochraceous, head and thorax with bluish green and reddish ochraceous markings.

**Figures 1–10.**
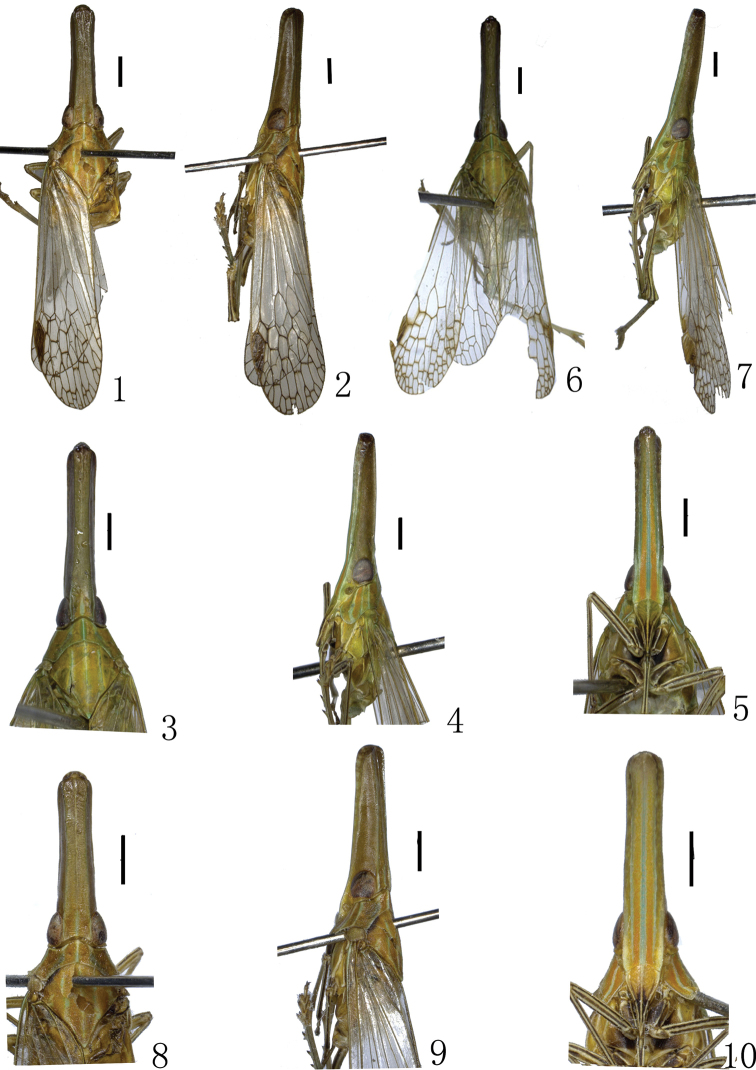
**1–5** Habitus of *Doryphorina conglobatus* sp. n. **6–10** *Doryphorina guizhouensis* sp. n. **1, 6** dorsal view **2, 7** lartral view **3, 8** dorsal view of head, pronotum and mesonotum **4, 9** lateral view of head and pronotum **5,10** ventral view of frons and clypeus. Scale bars: **1–10** = 1 mm.

**Figures 11–21.**
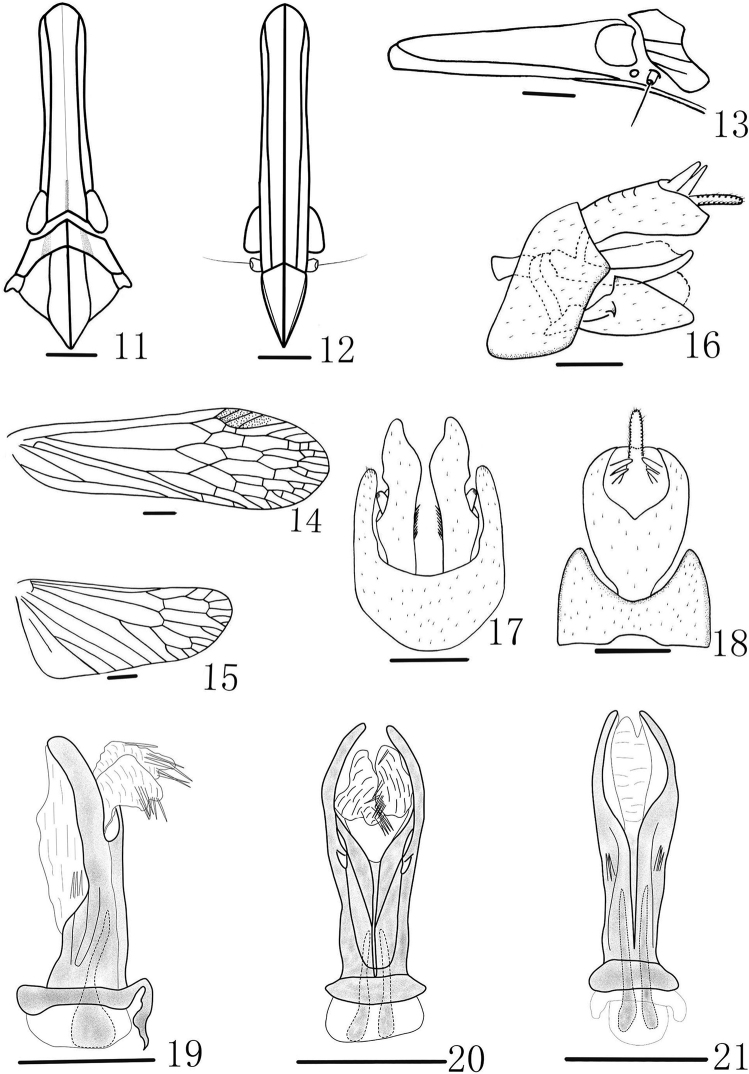
*Doryphorina conglobatus* sp. n. **11** Head and thorax, dorsal view **12** Frons and clypeus, ventral view **13** Head and pronotum, lateral view **14** Forewing **15** Hind Wing **16** Genitalia, lateral view **17** Pygofer and Gonostyles, ventral view **18** Pygofer and anal tube, dorsal view **19** Aedeagus, lateral view **20** Aedeagus, ventral view **21** Aedeagus, dorsal view. Scale bars: **11–15** = 1 mm, **11–16** = 0.5 mm.

Cephalic (Figs 1–3, 11, 13) process relatively robust, a little upturned, with ratio length to length of pronotum and mesonotum combined 1.6. Vertex (Figs 1, 3, 11) broad, media carina weakly, only distinct at apex and base, ratio of length to width between eyes 5.2. Frons (Figs 5, 12) elongate, intermediate carinae sub-parallel, nearly approaching frontoclypeal suture; median carina complete, length 5.3 times long than width. Pronotum (Figs 1–3, 11, 13) distinctly shorter than mesonotum in the middle line, median carina distinct, lateral carina obscure, only slightly present at base. Mesonotum (Figs 1, 2, 11) tricarinate, lateral carinae straight, nearly parallel. Forewings (Figs 1, 14) hyaline, with ratio length to maximum width 3.3; stigma distinct, with 4–5 cells. Legs moderately elongate, fore femora not flattened and dilated, without spine; hind tibiae with 5 lateral black-tipped spines and 7 apical black-tipped teeth.

**Male genitalia.** Pygofer (Fig. 16) wider ventrally than dorsally, posterior margin with a blunt process, ventral margin depressed to accommodate anal tube. Anal tube (Fig. 18) in dorsal view, with apex broader than base, the ventral margin with wrinkle. Gonostyles (Fig. 16) relatively small in lateral view, with apical margin not exceeding the apex of anal tube in lateral view, inner face with numerous setae in ventral view. Aedeagus (Fig. 19) with 1 pair of short endosomal processes, without extending from phallotheca. Phallobase (Figs 19–21) sclerotized and pigmented, with 2 pairs of apical membranous lobes: dorsal apical lobes (Fig. 19) long and slender, with 3 long spines at base; ventral lobes (Fig. 19) extending ventrally, with about 6 long spines each.

**Female.** Unknown.

#### Type material.

Holotype ♂, China: Shaanxi, Cuihuashan, 26 Aug. 2008, coll. Yujian Li (GUGC).

#### Distribution.

China (Shaanxi).

#### Diagnosis.

This species is similar to *Doryphorina subdeflexa* but can be distinguished from the later by phallobase with 2 pairs of apical membranous lobes, dorsal apical lobes (Fig. 19) long and slender, with 3 long spines at base (dorsal part with two pairs of dorsolateral apical lobes directed laterally, with 2–3 long apical spines in dorsal view in *Doryphorina subdeflexa*).

#### Etymology.

This new species is derived from the Greek word “*conglobatus*”, which indicate that the apical lobes of phallobase are connected.

### Doryphorina guizhouensis

Taxon classificationAnimaliaHemipteraDictyopharidae

Zheng, Yang & Chen sp. n.

http://zoobank.org/A2B8FC58-606F-4F69-917C-90FCD0E8D7C8

Figs 6–10 , 22–32

#### Measurement.

♂, BL: 17.7–17.9 mm; HL: 5.4–5.6 mm; HW: 1.6–1.7 mm; FWL: 10.5–10.9 mm.

#### Description.

Body greenish or greenish-ochraceous, marked with bluish green and reddish ochraceous on head and thorax.

**Figures 22–32.**
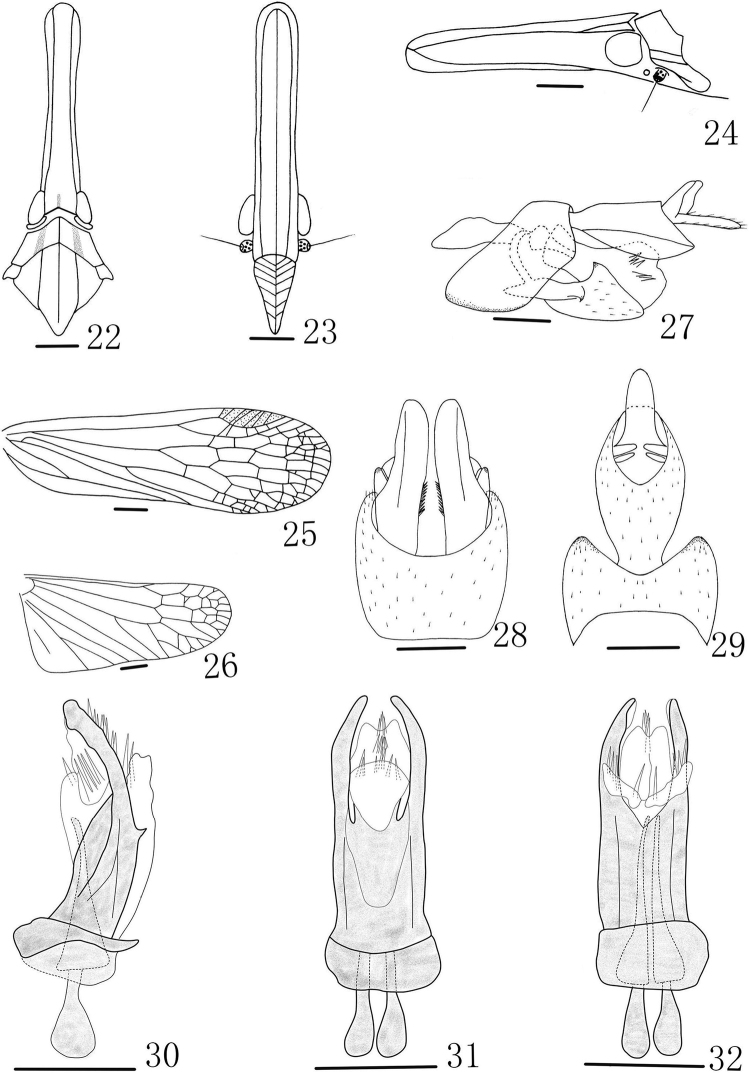
*Doryphorina guizhouensis* sp. n. **22** Head and thorax, dorsal view **23** Frons and clypeus, ventral view **24** Head and pronotum, lateral view **25** Forewing **26** Hind Wing **27** Genitalia, lateral view **28** Pygofer and Gonostyles, ventral view **29** Pygofer and anal tube, dorsal view **30** Aedeagus, lateral view **31** Aedeagus, ventral view **32** Aedeagus, dorsal view. Scale bars: **22–26** = 1 mm, **27–32** = 0.5 mm.

Cephalic (Figs 6–8, 22, 24) process relatively slender, a little upturned, with ratio length to length of pronotum and mesonotum combined 2.2. Vertex (Figs 6, 8, 22) broad, media carina weakly, only distinct at apex and base, ratio of length to width between eyes 6.3. Frons (Figs 10, 23) elongate, intermediate carinae sub-parallel, nearly approaching frontoclypeal suture; median carina complete, length 5.5 times long than wide. Pronotum (Figs 6–8, 22, 24) distinctly shorter than mesonotum medially, media carina distinct, lateral carina replaced by two pits. Mesonotum (Figs 6, 8, 22) tricarinate on disc, lateral carinae straight, nearly parallel. Forewings (Figs 6, 25) hyaline, with ratio length to maximum width 3.1; stigma distinct, with 4–5 cells. Legs moderately elongate, fore femora not flattened and dilated, without spine; hind tibiae with 5 lateral black-tipped spines and 7 apical black-tipped teeth.

**Male genitalia.** Pygofer (Fig. 27) wider ventrally than dorsally, posterior margin with a blunt process. Anal tube (Fig. 29) in dorsal view, the apex broader than base, the ratio of length to width about 1.8. Gonostyles (Fig. 27) relatively small in lateral view, shorter than the apex of anal tube in lateral view, inner face with numerous setae in ventral view. Aedeagus (Figs 30–32) with 1 pair of short endosomal processes, without extending from phallotheca. Phallobase (Figs 30–32) sclerotized and pigmented, with 2 pairs of apical membranous lobes: dorsal apical lobes (Fig. 31) long and slender, with 4 long apical spines each, ventral lobes (Fig. 31) with 2 pairs of apical lobes connected, not produced laterally, apex and base with about 16–19 long spines totally in ventral view.

**Female.** Unknown.

#### Type material.

Holotype ♂, China: Guizhou, Congjiang, 24 Jul. 2005, coll. Deyan Ge. Paratype, 1♂, China: Guizhou, Libo, Aug. 1997, coll. Zizhong Li (both in GUGC).

#### Distribution.

China (Guizhou).

#### Diagnosis.

This species is similar to *Doryphorina minor*, but can be distinguished from the later by phallobase ventral lobes (Fig. 31) with 2 pairs of apical lobes connected, not produced laterally, apex and base with about 16–19 long spines totally in ventral view (ventral part with two pairs of V-shaped elongate apical lobes, directed laterally and ventrally, each with 4–5 long spines in ventral view in *Doryphorina minor*).

#### Etymology.

This new species name refers to the type locality, Guizhou Province.

## Discussion

*Doryphorina conglobatus* sp. n. is similar to *Doryphorina subdeflexa* but can be distinguished from the later by phallobase lobes; *Doryphorina guizhouensis* sp. n. is similar to *Doryphorina minor* and *Doryphorina stali* but can be distinguished from *Doryphorina minor* by phallobase ventral lobes (Fig. 31); and can be distinguish from *Doryphorina stali* by phallobase dorsal lobes (Fig. 32); *Doryphorina conglobatus* sp. n. can be distinguished from *Doryphorina guizhouensis* sp. n. and *Doryphorina minor*, *Doryphorina stali* by the relatively robust cephalic process; the forewings without dull ochraceous spot near stigma. So, external features and phallobase lobes play an important role to identify of all male species in the genus.

The new species and all described species all distributed Oriental region. So far, this genus belongs to Oriental region.

## Supplementary Material

XML Treatment for Doryphorina

XML Treatment for Doryphorina conglobatus

XML Treatment for Doryphorina guizhouensis
